# Food Sources and Expenditures for Seafood in the United States

**DOI:** 10.3390/nu12061810

**Published:** 2020-06-17

**Authors:** David C. Love, Frank Asche, Zach Conrad, Ruth Young, Jamie Harding, Elizabeth M. Nussbaumer, Andrew L. Thorne-Lyman, Roni Neff

**Affiliations:** 1 Johns Hopkins Center for a Livable Future, Johns Hopkins University, Baltimore, MD 21202, USA; ruth.young@jhu.edu (R.Y.); jhardi14@jhu.edu (J.H.); enussba4@jhu.edu (E.M.N.); andrewtl@gmail.com (A.L.T.-L.); rneff1@jhu.edu (R.N.); 2 Department of Environmental Health and Engineering, Bloomberg School of Public Health, Johns Hopkins University, Baltimore, MD 21205, USA; 3 Institute for Sustainable Food Systems and School of Forest Resources and Conservation, University of Florida, Gainesville, FL 32611, USA; Frank.asche@ufl.edu; 4 Department of Industrial Economics, University of Stavanger, 4021 Stavanger, Norway; 5 Department of Health Sciences, William & Mary, Williamsburg, VA 23185, USA; zsconrad@wm.edu; 6 Department of International Health, Bloomberg School of Public Health, Johns Hopkins University, Baltimore, MD 21205, USA; 7 Center for Human Nutrition, Department of International Health, Bloomberg School of Public Health, Johns Hopkins University, Baltimore, MD 21205, USA

**Keywords:** diet, fish, salmon, shrimp, restaurant, food away from home, food at home, NHANES, retail, seafood

## Abstract

The aim of this study was to explore United States (U.S.) seafood consumption patterns, food sourcing, expenditures, and geography of consumption. We analyzed seafood intake and food sourcing using the National Health and Nutrition Examination Survey (NHANES) cycles 2007–2008 to 2015–2016 for US adults ≥19 years old (*n* = 26,743 total respondents; *n* = 4957 respondents consumed seafood in the past 24 h). Seafood expenditures were extrapolated by combining NHANES with three other public datasets. U.S. adults consumed 63% of seafood (by weight) at home. The top sources of seafood (by weight) were food retail (56%), restaurants (31%), and caught by the respondent or someone they know (5%). Sixty-five percent of consumer expenditures for seafood were at restaurants and other “away from home” sources while 35% were at retail and other “at home” sources. Slightly less than half of overall U.S. food expenditures are “away from home,” which is much lower than for seafood, suggesting that consumers have very different spending habits for seafood than for an aggregate of all foods.

## 1. Introduction

United States (U.S.) consumers receive the majority of their protein from terrestrial animals [1], however, seafood consumption has been linked with numerous health benefits, including reduced risk of coronary heart disease, ischemic stroke, and cardiac death [2,3]. Federal dietary guidelines recommend that adults consume at least 227 g/week (8 oz/week) of seafood based on a 2000 kcal diet (for reference, one seafood serving is 85 g or 3 oz, cooked wt) [4]. Only 10% to 20% of U.S. consumers meet the federal Dietary Guidelines [5].

The current U.S. seafood supply provides 140 g/week (4.9 oz/week) per person, a level that has remained relatively constant for three decades [6,7]. Market growth in terms of amount consumed is significant [6,7], but mainly reflects population growth. Analyses of trade data indicate that the five top species consumed by Americans are shrimp, salmon, canned tuna, catfish/pangasius, and tilapia, which jointly comprise 70–80% of the U.S. seafood supply [6]. Focus has shifted from wild capture to farm-raised products, and there is an increasing reliance on imports as U.S. production has been stable since the mid-1980′s [6].

Americans spend $102 billion each year on seafood, predominantly at food service venues (e.g., restaurants, institutional food) [7], however, this finding has not been independently verified. Retail scanner data can explain market trends among grocery shoppers by product, region, and season [8,9,10], but these analyses have been limited to frozen and canned seafood and do not include fresh seafood. Consumers note preferences to purchase familiar types of seafood, which limits the demand for diverse fish types [11]. Economies of scale in the supply chain accentuate this narrowing [12]. Consumer purchasing behavior is multi-faceted, as are the drivers and barriers that affect seafood consumption [13]. Price, convenience, product quality, taste, culture, preferences, and habits, as well as origin, production methods, and labeling are each important [13]. Consumers also say they are willing to spend more for seafood with environmental or social certifications [14,15], however, the complex messaging around these issues can confuse consumers [16].

Several key knowledge gaps exist around seafood consumption. There is little detailed information about consumer habits when sourcing seafood. For example, is seafood more often consumed at lunch or dinner? Do consumers purchase some species more often at supermarkets or restaurants? There is also incomplete information about the distribution of seafood consumption within the U.S. The present study seeks to describe national seafood consumption patterns, food sourcing and expenditures using multiple, publicly available data sources. Dietary habits can serve as a proxy for purchasing behaviors and provide a more nuanced understanding of consumer seafood choices than was previously available. This study can also inform government agencies and healthcare professionals that may wish to change Americans’ seafood consumption or shift purchasing habits.

## 2. Materials and Methods

### 2.1. Dietary Data

The National Health and Nutrition Examination Survey (NHANES) is a nationally representative survey of Americans that is administered by the U.S. government. NHANES uses a complex, multistage, probability sampling design that continuously samples approximately seven thousand non-military, non-institutionalized Americans annually, in order to obtain a nationally representative sample [17]. Data are released in two-year cycles. One component of NHANES is a 24-h dietary recall survey called What We Eat In America (WWEIA), which is administered by the U.S. Departments of Agriculture (USDA) and Health and Human Services (HHS) [18]. NHANES and WWEIA (which we will collectively refer to as NHANES) collect detailed information about food intake, sourcing, time and location of consumption, the nutritional content of foods, and supporting demographic information.

### 2.2. NHANES Seafood Analysis

We analyzed the NHANES day-1 dietary recall dataset for adults (≥19) respondents over five survey cycles (2007–2008, 2009–2010, 2011–2012, 2013–2014, and 2015–2016), which contained 26,743 total adult respondents, including 4957 individuals who reported consuming seafood (i.e., fish, crustaceans, mollusks, other aquatic animals) in the prior day (henceforth: “seafood consumers”). NHANES data were joined with the USDA Food Patterns Equivalents Database [19], which converts foods consumed by NHANES respondents into grams by food group—in this case grams of seafood. 

We assessed per capita seafood consumption in relation to several sociodemographic status variables: sex; age (19–30, 31–50, 51–70, +71); and income (above or below the 185% poverty threshold). These variables and break-points were chosen to better compare with previous NHANES seafood analyses [5]. We then analyzed seafood consumption by the location where a meal was purchased (variable name: “DR1FS”), the location where a meal was consumed (variable name: “DR1_040Z”), and the meal type (variable name: “DR1_030Z”) among seafood consumers. We used established definitions for food source created by the USDA: “Food at home” includes food obtained at grocery store or seafood caught by the consumer or someone known to them; and “food away from home” includes food obtained from a restaurant, institution, or school (Supplementary Materials Table S1) [20]. 

We explored seafood species intake by food source to better understand where Americans purchase the seafood species they consume. We accessed per capita seafood supply from the National Marine Fisheries Service [7] and National Fisheries Institute [21], and then compared per capita supply to per capita consumption. 

### 2.3. Seafood Expenditure by Food Source 

We estimated consumer spending by food source using previously described methods [22]. Briefly, we linked the NHANES database to the Center for Nutrition Policy and Promotion Food Prices Database, Consumer Price Index, and USDA National Household Food Acquisition and Purchase Survey (as described in [22]). Each food reported consumed by NHANES participants was linked with a food commodity in the USDA Loss-Adjusted Food Availability Database, which provides data on the amount of food lost and wasted at each point in the food system. This method also accounts for the consumer cost of seafood waste and inedible portions, as described by others [23].

### 2.4. Database Management and Statistical Analysis

Data were analyzed in R Studio (v1.2). We accounted for the complex sampling design within NHANES using primary sampling units, strata, and a 5-year weighted average variable to construct unbiased national estimates of seafood consumption. Two-sample *t*-tests were used to compare variables, such as fish consumption by gender. Survey-weighted generalized linear models were fit to compare three or more variables such as age classes. All data analyses were carried out in R Studio using the R statistical language and the *survey* and *srvyr* packages.

### 2.5. Geography of Seafood Consumption

We used ArcGIS (v10.7, Redlands, CA, USA) to map seafood consumption by U.S. county. We achieved this by determining the per capita consumption (g/day raw weight, edible portion) by county [24] and then multiplying by the county population [25] to get tons of seafood consumed per county per year. Per capita consumption by region and coastal vs. non-coastal areas was reported by the U.S. Environmental Protection Agency using NHANES analyses and modeling (Supplementary Materials Table S4) [24]. Log scale was used to account for the wide range in quantities consumed over space. The map uses WSF 1984 Web Mercator (auxiliary sphere) coordinate system and the county boundaries on the map are derived from the 2017 U.S. County TIGER/Line Shapefile.

## 3. Results

### 3.1. Per Capita Seafood Consumption

Average seafood consumption per capita was 18 g in the past 24-h across adult seafood consumers and non-consumers (Table 1). Among seafood consumers (respondents who consumed seafood in the past 24 h), average seafood consumption was 106.9 g (3.7 oz) (Table 1). Seafood consumption amounts were larger among men compared to women, those of middle age (31–50 y/o) compared to other ages, and individuals with higher incomes versus lower. Lower income was set at the 185% poverty threshold, which for a family of four would be $38,000 to $45,000 annual income during the study period.

### 3.2. Food Sourcing of Seafood

Sixty-one percent of seafood intake (by weight) was obtained from food sources consistent with USDA’s definition of “food at home” and 39% from food sources within the “food away from home” category (Table 2). The top five most commonly reported sources of seafood (by weight) were stores (i.e., grocery stores, 56%), restaurants (31%), a gift (5%), fish caught by you or someone you know (5%), or institutional food (2%), jointly accounting for nearly 98% of all seafood sources. 

We found good agreement between questions related to food sourcing and the location where the meal was consumed. Overall, 63% of seafood by weight was eaten at home, which is consistent with 61% of seafood by weight from “food at home” sources. When exploring the “food at home” category, 88% of seafood from these sources was eaten at home. Twenty-five percent of seafood from restaurants and other “food away from home” locations was eaten at home, which could be restaurant take-out or meal delivery. 

The average per capita seafood meal size was 96.0 g (3.4 oz), which was 13% more than the U.S. recommended serving size for cooked seafood (85 g or 3 oz) [4,26]. The average meal size from food purchased at retail stores was 97.1 g (or 3.4 oz) and restaurants was 90.8 g (or 3.2 oz). Self-caught fish meals (170.0 g) were significantly larger than average seafood meal sizes from all sources.

Next, we explored when seafood was consumed (breakfast, lunch, dinner, snack) and where these meals were purchased and consumed (Table 2). Seafood was primarily consumed at dinner (62% by weight) with the remainder at lunch (29%), snack (4%) or breakfast (3%). Based on quantity consumed per meal, seafood portions were 30% larger at dinner than lunch. Seafood dinners were primarily consumed at home, while seafood lunches were more often consumed away from home at restaurants and other venues. 

### 3.3. Seafood Species Consumed 

By calculating the total quantity consumed by Americans, the species consumed in the greatest amounts (from most to least) were salmon, shrimp, canned tuna, “fish”, tilapia, and catfish (Table 3). Interestingly, there were preferences to purchase some species at retail stores (e.g., tilapia, canned tuna, and salmon) and other species at restaurants and other “away from home” venues (e.g., crab, shrimp, cod). To provide another perspective on sourcing we analyzed responses to a different question: “was the meal consumed at home?” and found good agreement with food sourcing. Food sourced from retail was largely eaten at home, and food sourced from restaurants and other venues was eaten away from home. The increased share of home consumption (compared to sourcing for foods often purchased at restaurants) may be due to restaurant take-out or meal delivery. We present consumption according to species and food source for adults and all ages in Supplementary Materials Tables S2 and S3.

### 3.4. Intake Versus Supply 

We compared seafood supply reported by National Marine Fisheries Service (NMFS) to seafood consumption from in this study (Table 4). These comparisons were made both on a per-capita basis (lbs/yr, which is consistent with the supply data as provided) and by the rank order by species. Total per capita consumption was 11.7 lb/yr (5.3 kg/yr) edible portion, cooked wt, which is 24% less than the U.S. seafood supply (15.3 lb/yr or 6.9 kg/yr, edible portion, raw wt) [1]. Supply and consumption rates were proportional across species, suggesting NMFS and NHANES data sets are overall in agreement. Consumption frequencies generally predicted the rank order of species available in the U.S. seafood supply, other than shrimp and catfish supplies, which were disproportionately high compared to consumption. 

### 3.5. Seafood Expenditures by Food Source 

We modeled seafood expenditures by food source (Table 5). We found “food at home” and “food away from home” expenditures were 35% and 65% of the retail value, respectively, which is a similar ratio to previous estimates by NMFS. Similar results using two different sets of methods and datasets suggest that the findings are reliable.

### 3.6. Geography of Seafood Consumption

Seafood consumption is not evenly spread across the U.S. Living away from the coast (>25 mi), regardless of the region in the U.S., was associated with a 5 g/day (0.18 oz/day) drop in seafood consumption among adults (Supplementary Materials Table S4, Figure S1). When combining coastal status and region, the lowest consumption rates were among inland Midwest (12.4 g/day; 0.44 oz/day) and inland Great Lakes (14.6 g/day; 0.52 oz/day) adults, and the highest consumption rates were among coastal Northeast (24.5 g/day;0.86 oz/day) and coastal Pacific (22.1 g/day; 0.78 oz/day) adults.

**Figure 1:**
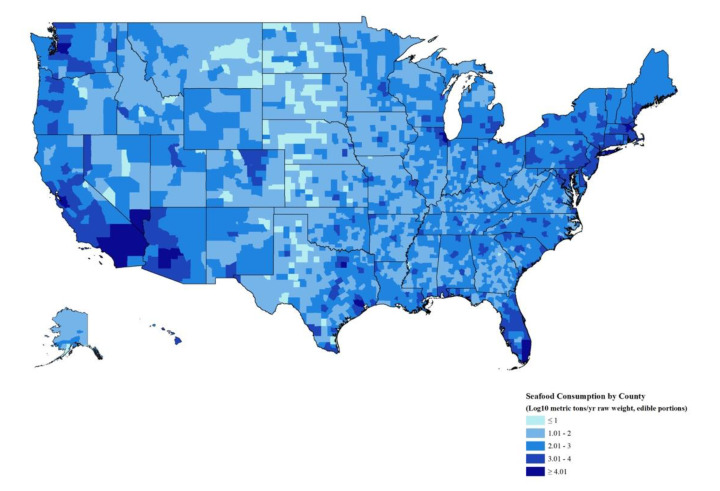
Seafood consumption by county (log_10_ metric tons/yr raw weight, edible portion).

To better understand the spatial variability, we multiplied per capita consumption rates (Supplementary Materials Table S4, Figure S1) by population density to obtain total seafood demand by county (Figure 1). As one might expect, urban centers consumed more seafood than rural areas. This was true regardless of whether the city was near to or far from the coast (Figure 1). Somewhat surprisingly, demand in non-urban coastal regions was moderate to low, which indicates that population density is a stronger indicator of demand than coastal proximity. 

## 4. Discussion

This study uses nationally representative dietary intake data (i.e., NHANES) linked with other sources to provide a more nuanced picture of U.S. seafood sources and expenditures than has previously been described in other NHANES seafood studies [5,27,28,29,30]. Overall, seafood consumption levels were greater among men, those of middle age, and those with higher incomes compared with women, those of other ages, and those with lower incomes, which has been noted previously [5]. Seafood consumers obtained 39% of their seafood by weight from restaurants and other “away from home” venues, however, these food sources accounted for a disproportionate (65%) share of consumer expenditures [31,32]. Seafood consumption rates generally tracked that of the U.S. seafood supply, and the difference between them was likely due to water loss during cooking and food waste. The largest regions for seafood consumption were in coastal and inland cities. Although per-capita consumption is greater in coastal areas, overall seafood demand in non-urban coastal areas (represented as tons per county) was not as high as might have been expected. 

One of our main findings relates to sourcing seafood at food service venues such as restaurants. Over the past half-century, the general trend among consumers is to cook less at home and eat out more [31,32,33]. It is estimated that Americans consume 28–35% of calories away from home [32]. We found that on average 39% of total seafood intake by weight was purchased at restaurants and other food service venues, and that the rate could be as high as 50% to 60% for some species such as crab, catfish, cod, and shrimp. Seafood meals at restaurants were similar in size to federally recommended serving sizes for seafood [4,26]. Seafood is more likely to be purchased at restaurants and other food service venues compared to other food products, although our by-weight seafood analysis cannot be directly compared to the by-energy caloric estimate. 

On a per dollar basis, seafood expenditures away from home are notable. Today slightly less than half (48%) of overall U.S. food expenditures are away from home. By contrast, seafood expenditures at restaurants and other food service venues represent two-thirds of consumers’ seafood dollars. Consumers, therefore, have very different spending habits for seafood than for an aggregate of all foods. 

Consumers preferred buying some products from retailers and others from restaurants. Shrimp and salmon are the top two seafood products consumed in the U.S. [6] and we found that (by weight) salmon was more often purchased at retail outlets like grocery stores, while shrimp was more often purchased at restaurants and other food service venues. These food sourcing preferences may be related to consumers’ perceived ability or comfort to cook certain species at home or the availability of products at different venues [34,35]. The average salmon meal was twice the quantity of a shrimp meal, which may be related to how shrimp is used as an ingredient in dishes while salmon is often a center of the plate protein. The time of day can also influence consumption sourcing; seafood eaten at dinner is more often purchased from retail outlets, while seafood lunches were more often purchased from restaurants and other “away from home” venues. Certain products such as crabs, lobster, scallops, and snails were purchased more at restaurants than retail outlets, perhaps because they are perceived as a luxury item or require special cooking methods compared to everyday food. 

Previous analyses of household food expenditure at the national level have not accounted for the cost of food waste and inedible portions, as well as the important price differences between foods consumed at home and foods consumed away from the home. In the present study, we utilize nationally representative food expenditure data from a recent study by Conrad [22] that accounted for these important aspects of household food expenditure, while also using established methods to account for food price inflation to provide contemporary estimates (2016 U.S. dollars). Our findings on seafood expenditures are mostly within a few percentage points of the NMFS expenditure estimates. Differences in dollar expenditures between NMFS and our findings could be due to Conrad’s methods of accounting for the cost of food wasted at the store, inedible portions, and differences in “food away from home” costs. Given that NMFS model is built from supply-side data and ours is based on consumer intake and consumer price data, the similarity is striking and adds validity to the findings. 

Several strengths and limitations are worth noting. NHANES has distinct advantages as a data source beyond its overall sampling quality and level of methodological rigor. First, we provide per capita estimates of intake, which is an improvement over previous estimates of per capita supply used as a proxy for intake [21]. Second, these data also include self-caught seafood, either as recreational or subsistence fishing, which is generally missing from per capita supply. Third, NHANES provides a rich individual-level dataset (age, sex, income, diet, health) which provides the opportunity to better understand demographic trends for future analyses. One limitation is the way seafood intake is collected. NHANES does not have response options for some species, most notably Alaska pollock, and other species were instead reported as “fish” or “seafood.” We used a single day of dietary intake, which is a cross sectional snapshot and may not reflect the population intake in the long term. While the mean intake from a single day will be accurate for the population, the standard error bars are wider than they would be using multiple days of intake because we have not accounted for random within-person variability [36]. Lastly, self-reported data have limitations in recall that might differ across types of seafood, portion size estimation, and the potential for overreporting seafood consumption due to social desirability bias.

## 5. Conclusions 

This study addresses key knowledge gaps at the consumer-level about sourcing practices and expenditures for seafood using several nationally representative and publicly available datasets. We found that on average 39% of total seafood intake by weight was eaten away from home, mostly at restaurants, and it could be as high as 50% to 60% for some species, while 65% of consumer expenditures on seafood was at “food away from home” venues. U.S. adult expenditures on seafood at “away from home” venues are larger than the national trends for all foods. The analysis has broader implications because it identifies populations and locations where seafood consumption is particularly low and can inform government agencies and healthcare professionals that may wish to change Americans’ seafood consumption or shift purchasing habits.

Currently, most Americans do not meet national dietary recommendations for seafood as described in the Dietary Guidelines for Americans 2015–2020 [5]. Based on our findings, interventions to promote seafood consumption focusing only on at-home cooking would miss an important source for the American seafood diet, namely fast food and other chain restaurants. Food sourcing decisions have an impact on health; for example, cooking at home is typically healthier than eating out [37,38,39]. Further work is needed to understand the nutritional content of seafood at restaurants compared to home-cooked meals and to better align restaurant meals with recommendations in the Dietary Guidelines for Americans. 

## Figures and Tables

**Table 1:** Per capita seafood intake in United States adults (≥19) in the past 24 h (National Health and Nutrition Examination Survey (NHANES) 2007 to 2016).

Category	All NHANES Respondents (Equal to US Per Capita Intake)		NHANES Seafood Consumers (Seafood in Past 24 h)	
	*n*	Mean ± SE (g/day Cooked Weight)	*n*	Mean ± SE (g/day Cooked Weight)
**All adults**	26,743	18.0 ± 0.8	4957	106.9 ± 2.9
Men (≥19)	13,046	20.4 ± 1.1 ^a^	2355	125.5 ± 4.4 ^a^
Women (≥19)	13,697	15.8 ± 0.7	2602	90.7 ± 2.5
**Age (years)**				
19–30	5521	14.9 ± 1.3	855	106.0 ± 6.6
31–50	8889	18.9 ± 1.2 ^a^	1706	111.5 ± 4.8 ^a^
51–70	8386	19.8 ± 1.2 ^a^	1708	106.3 ± 4.6
>70	3947	16.5 ± 1.1 ^a^	688	95.3 ± 3.7 ^a^
**Income**				
0–185% poverty threshold	11,277	16.8 ± 0.9	1801	104.5 ± 3.2
>185% poverty threshold	12,947	18.8 ± 0.9 ^a^	2680	115.0 ± 4.5 ^a^

^a^ Values are significantly different (*p* < 0.05). For age, the comparator was age group 19–30.

**Table 2:** Meal type, food source, and location of meal in United States adult seafood consumers (≥19) in the past 24 h (NHANES 2007 to 2016).

Category	Seafood Meals Consumed in Past 24 h by NHANES Respondents (*n*)	Seafood Consumed		Was the Seafood Meal Consumed at Home? (% yes)
		Percent of Total, by Source or Meal Type	Mean ± SE (g/meal, Cooked Weight)	
**Overall**	5604 ^b^	100%	96 ± 2.7	63%
**Food Source** ^a^				
Home	3204	61%	100 ± 3.3	88%
Away from Home	2400	39%	90 ± 3.7	25%
**Food Source, breakout**				
Store	3062	56%	97 ± 3.2	89%
Restaurant	1844	31%	91 ± 4.4	25%
Gift	372	5%	80 ± 5.8 ^d^	27%
Self-caught	114	5%	170 ± 25 ^d^	82%
Institutional food	113	2%	97 ± 12	11%
Other ^c^	47	1%	108 ± 19	4%
Bar, sports, recreation	23	1%	102 ± 14	71%
Soup kitchen, community food program	25	0.3%	96 ± 20	70%
**Meal**				
Dinner	3042	62%	107 ± 3.4	69%
Lunch	1826	29%	83 ± 3.1	47%
Snack	333	4%	63 ± 4.6	75%
Breakfast	217	3%	88 ± 15	79%
Other ^e^	188	2%	114 ± 12	63%

^a^ At home (FAH: food obtained from a food retail (i.e., supermarkets), mail order, or caught by you or someone you know); away from home (FAFH: food from all other sources including restaurants, schools, institutions, street food, etc.). FAH and FAFH variables described by the USDA in [20] and Supplementary Materials Table S1; ^b^ Some respondents consumed more than one seafood meal in a 24-h period, which explains why the overall ‘n’ is larger than in Table 1. ^c^ Other: vending machine, street vendor, fund raiser, common coffee pot or snack tray, self-reported “don’t know”; ^d^ By weight of seafood consumed; values are significantly different (*p* < 0.05) ^e^ Other: unnamed meal and continuous eating.

**Table 3:** Top seafood species consumed by United States adult seafood consumers (≥19) (NHANES 2007 to 2016).

Species Group	Seafood Meals Consumed in Past 24 h by NHANES Respondents (*n*)	Total Consumed in U.S. ± SE (Metric Tons/day Cooked Weight) ^a^	Mean Consumed Per Meal ± SE (g/day Cooked Weight) ^b^	Food Source (% Home/% Away from Home) ^a^	Was the Seafood Meal Consumed at Home? (% yes)
Salmon	586	625 ± 68	111 ± 6.7	71%/29%	70%
Shrimp	1536	589 ± 46	55 ± 2.8	45%/55%	52%
Canned Tuna	718	499 ± 37	71 ± 2.4	82%/18%	74%
“Fish” ^c^	871	438 ± 30	84 ± 3.7	51%/49%	58%
Tilapia	344	391 ± 57	166 ± 13	84%/16%	77%
Catfish	235	213 ± 52	157 ± 19	49%/51%	58%
Cod	174	162 ± 23	129 ± 9.0	45%/55%	54%
Crab	260	148 ± 25	72 ± 7.3	36%/64%	49%
“Seafood” ^c^	329	89 ± 10	38 ± 2.6	55%/45%	66%
Flounder	96	96 ± 18	129 ± 14	54%/46%	58%

^a^ By total weight of seafood consumed among all seafood consumers. At home, away from home defined in Table 2. ^b^ Per individual consuming that species group in the past 24 h; ^c^ If species group was not provided, the food was assigned either “fish”, “seafood”, or “shellfish”.

**Table 4:** Per capita supply (National Marine Fisheries Service (NMFS)) and consumption (NHANES) of top seafood species (2007 to 2016).

Species	Supply ^a^		Consumption ^b^	
	Per Capita (Mean lb/year Raw Edible Weight)	Percent of Total	Per Capita (Mean lb/year Cooked Weight)	Percent of Total
Shrimp	4.00	26%	1.67	14%
Salmon	2.23	15%	1.70	14%
Canned tuna	2.46	16%	1.46	12%
“Fish” ^c^	--	--	1.36	12%
Tilapia	1.32	9%	1.13	10%
Catfish ^d^	1.31	9%	0.57	5%
Alaska pollock	1.23	8%	n/a ^e^	n/a ^e^
Crab	0.56	4%	0.45	4%
Cod	0.53	3%	0.42	4%
“Seafood” ^c^	--	--	0.27	3%
Clams	0.37	2%	0.13	1%
Total ^f^	15.3	100%	11.7	100%

^a^ Per capita supply is reported as the total raw edible weight divided by the US population (NMFS/NFI, 2007–2016). The government provides these values as lbs so we maintain the same units for comparison purposes. Data sources: [7,21]; ^b^ Per capita consumption reported as cooked weight (NHANES, 2007–2016); ^c^ “Fish”, “shellfish”, or “seafood” were reported if species group was not provided; ^d^ Catfish and pangasius summed for total catfish supply; ^e^ N/a = not available. Alaska pollock not available as a species to select in NHANES; ^f^ Total is greater than the sum of the rows due to additional seafood species not listed.

**Table 5:** Seafood expenditure by food source modeled using supply (NMFS) and consumption data (NHANES) (2007–2016).

Food Source	NMFS ^b^ Seafood Expenditures		NHANES Seafood Expenditures	
	Mean ± SE (Million USD)	Percent of Total ± SE	Mean ± SE ^c^ (Million USD)	Percent of Total
Home ^a^	26 ± 0.82	32%	111 ± 6.1	35%
Away from home ^a^	55 ± 1.3	68%	209 ± 11.0	65%
Total	81 ± 2.1	100%	310 ± 12.6	100%

^a^ Home and away from home defined in Table 1; ^b^ data source: [7,21]; ^c^ Adjusted to 2016 US Dollars.

## Supplementary Materials

The following is available online at https://www.mdpi.com/2072-6643/12/6/1810/s1, Table S1. U.S. Department of Agriculture “food at home” (FAH) and “food away from home” (FAFH) codes. Table S2. Top seafood species consumed by United States adult seafood consumers (≥ age 19) by food source (NHANES 2007 to 2016). Table S3. Top seafood species consumed by United States seafood consumers (all ages) by food source (NHANES 2007 to 2016). Table S4. Usual fish consumption rates (g/day raw weight, edible portion) of total fish and shellfish. 50th percentile estimates (range: 95% CI). Figure S1. Seafood consumption rates (g/day, raw weight, edible portion) by county for each coastal/inland region described in Table S4.
