# Supplementary material for: Food Sources and Expenditures for Seafood in the United States
Source: Nutrients. 2020 Jun 17;12(6):1810. doi: 10.3390/nu12061810 (PMC7353403; doi:10.3390/nu12061810)
Supplement: Supplementary file 1 [file nutrients-12-01810-s001.pdf]

Supplementary

Food Sources and Expenditures for Seafood in the United States

David C. Love [1,2,*], Frank Asche [3,4], Zach Conrad [5], Ruth Young [1,6], Jamie Harding [1,2], Elizabeth M. Nussbaumer [1,2], Andrew L. Thorne-Lyman [1,2,7] and Roni Neff [1,2]

[1] Johns Hopkins Center for a Livable Future, Johns Hopkins University, Baltimore, MD 21202, USA; ruth.young@jhu.edu (R.Y.); jhardi14@jhu.edu (J.H.); enussba4@jhu.edu (E.N.); andrewtl@gmail.com (A.L.T.-L.); rneff1@jhu.edu (R.N.)
[2] Department of Environmental Health and Engineering, Bloomberg School of Public Health, Johns Hopkins University, Baltimore, MD 21205, USA
[3] Institute for Sustainable Food Systems and School of Forest Resources and Conservation, University of Florida, Gainesville, FL 32611, USA; Frank.asche@ufl.edu
[4] Department of Industrial Economics, University of Stavanger, Stavanger, Norway
[5] Department of Health Sciences, William & Mary, Williamsburg, VA 23185, USA; zsconrad@wm.edu
[6] Department of International Health, Bloomberg School of Public Health, Johns Hopkins University, Baltimore, MD 21205, USA
[7] Center for Human Nutrition, Department of International Health, Bloomberg School of Public Health, Johns Hopkins University, Baltimore, MD 21205, USA
* Correspondence:dlove8@jhu.edu

List of Supplementary Tables and Figures:

Table S1. US Department of Agriculture "food at home" (FAH) and "food away from home" (FAFH) codes. [1].

Table S2. Top seafood species consumed by United States adult seafood consumers (≥ age 19) by food source (NHANES 2007 to 2016).

Table S3. Top seafood species consumed by United States seafood consumers (all ages) by food source (NHANES 2007 to 2016).

Table S4. Usual fish consumption rates (g/day raw weight, edible portion) of total fish and shellfish. 50th percentile estimates (range: 95% CI). [2].

Figure 1. Seafood consumption rates (g/d, raw weight, edible portion) by county for each coastal/inland region described in Table S4

Table S1. US Department of Agriculture "food at home" (FAH) and "food away from home" (FAFH) codes. [1].

Code	Description	FAH or FAFH
1	Store - grocery/supermarket	FAH
2	Restaurant with waiter/waitress	FAFH
3	Restaurant fast food/pizza	FAFH
4	Bar/tavern/lounge	FAFH
5	Restaurant no additional information	FAFH
6	Cafeteria NOT in a K-12 school	FAFH
7	Cafeteria in a K-12 school	FAFH
8	Child/Adult care center	FAFH
9	Child/Adult home care	FAFH
10	Soup kitchen/shelter/food pantry	FAH if eaten at home; otherwise FAFH
11	Meals on Wheels	FAFH
12	Community food program - other	FAH if eaten at home; otherwise FAFH
13	Community program no additional information	FAH if eaten at home; otherwise FAFH
14	Vending machine	FAFH
15	Common coffee pot or snack tray	FAFH
16	From someone else/gift	FAFH
17	Mail order purchase	FAH
18	Residential dining facility	FAFH
19	Grown or caught by you or someone you know	FAH
20	Fish caught by you or someone you know	FAH
24	Sport, recreation, or entertainment facility	FAFH
25	Street vendor, vending truck	FAFH
26	Fundraiser sales	FAFH
27	Store - convenience type	FAFH
28	Store - no additional info	FAFH
91	Other, specify	FAFH
99	Don't know	FAFH
.	Missing	FAFH

Table S2. Top seafood species consumed by United States adult seafood consumers (≥ age 19) by food source (NHANES 2007 to 2016).

**Food Source (metric tons/day ± SE)**	**Salmon**	**Shrimp**	**Canned Tuna**	**Fish**	**Tilapia**	**Catfish**	**Cod**	**Crab**	**Flounder**	**Seafood**
Store	412 ± 54	251 ± 27	406 ± 34	216 ± 19	314 ± 52	83 ± 32	69 ± 17	46 ± 12	44 ± 11	49 ± 8
Restaurant	136 ± 25	272 ± 26	64 ± 12	159 ± 18	35 ± 7	82 ± 15	67 ± 14	81 ± 16	41 ± 14	35 ± 6
Self-caught	27 ± 25	13 ± 6	-	4 ± 2	14 ± 8	20 ± 13	4 ± 2	4 ± 3	9 ± 6	-
Gift	26 ± 9	29 ± 5	14 ± 3	45 ± 10	15 ± 8	9 ± 2	7 ± 1	8 ± 5	-	3 ± 2
Institution	17 ± 10	10 ± 4	6 ± 3	4 ± 1	11 ± 6	5 ± 3	7 ± 5	2 ± 2	2 ± 1	2 ± 2
Bar, sports, rec facility	1.3 ± 1.3	6 ± 3	1 ± 1	4 ± 3	-	11 ± 10	2 ± 2	-	0.5 ± 0.5	0.1 ± 0.1
Soup kitchen, com food prog	0.7 ± 0.7	0.8 ± 0.8	5 ± 3	2 ± 1	-	0.3 ± 0.2	2 ± 2	-	-	-
Other	1 ± 0.9	7 ± 6	4 ± 3	4 ± 1	2 ± 2	2.3 ± 1.6	5 ± 4	2 ± 2	0.5 ± 0.5	1 ± 1
Don't know	3 ± 2	0.5 ± 0.4	-	2 ± 1	0.9 ± 0.6	-	-	1 ± 1	-	0.1 ± 0.1
**Food Source (% by source)**	**Salmon**	**Shrimp**	**Canned Tuna**	**Fish**	**Tilapia**	**Catfish**	**Cod**	**Crab**	**Flounder**	**Seafood**
Store	66%	43%	81%	49%	80%	39%	43%	32%	45%	54%
Restaurant	22%	46%	13%	36%	9%	39%	41%	57%	43%	39%
Self-caught	4%	2%	0%	1%	3%	10%	2%	3%	9%	0%
Gift	4%	5%	3%	10%	4%	4%	4%	5%	0%	3%
Institution	3%	2%	1%	1%	3%	2%	5%	1%	2%	3%
Bar, sports, rec facility	0%	1%	0%	1%	0%	5%	1%	0%	0%	0%
Soup kitchen, comm food prog	0%	0%	1%	0%	0%	0%	1%	0%	0%	0%
Other	0%	1%	1%	1%	1%	1%	3%	1%	1%	1%
Don't know	1%	0%	0%	0%	0%	0%	0%	1%	0%	0%
TOTAL	100%	100%	100%	100%	100%	100%	100%	100%	100%	100%

Table S3. Top seafood species consumed by United States seafood consumers (all ages) by food source (NHANES 2007 to 2016).

Food Source (metric tons/day ± SE)	Salmon	Shrimp	Canned Tuna	Fish	Tilapia	Catfish	Cod	Crab	Flounder	Seafood
Store	438 ± 55	278 ± 28	459 ± 36	265 ± 22	355 ± 52	86 ± 33	79 ± 18	52 ± 13	51 ± 12	61 ± 9
Restaurant	140 ± 25	297 ± 26	70 ± 13	182 ± 21	42 ± 8	84 ± 15	68 ± 14	88 ± 16	42 ± 14	39 ± 6
Self-caught	29 ± 25	15 ± 7	0.1 ± 0.1	7 ± 3	14 ± 8	23 ± 13	4 ± 2	4 ± 4	9 ± 6	-
Gift	28 ± 9	34 ± 6	18 ± 4	52 ± 10	16 ± 8	11 ± 3	7 ± 1	9 ± 5	-	-
Institution	18 ± 10	11 ± 4	9 ± 3	13 ± 3	12 ± 6	5 ± 3	10 ± 5	2 ± 1	2 ± 1	2 ± 2
Bar, sports, rec facility	1 ± 1	6 ± 3	1 ± 1	4 ± 3	-	11 ± 10	2 ± 2	-	1 ± 1	0.08 ± 0.08
Soup kitchen, com food prog	1 ± 1	1 ± 1	6 ± 3	2 ± 1	-	0.3 ± 0.2	2 ± 2	-	-	-
Other	1 ± 1	7 ± 6	4 ± 3	4 ± 2	2 ± 2	2 ± 2	5 ± 4	2 ± 2	1 ± 1	1 ± 1
Don't know	4 ± 3	1 ± 0.4	-	2 ± 1	1 ± 1	0.04 ± 0.04	-	1 ± 1	-	0.05 ± 0.05
**Food Source (%)**	**Salmon**	**Shrimp**	**Canned Tuna**	**Fish**	**Tilapia**	**Catfish**	**Cod**	**Crab**	**Flounder**	**Seafood**
Store	66%	43%	81%	50%	80%	39%	45%	33%	49%	57%
Restaurant	21%	46%	12%	34%	9%	38%	39%	56%	40%	37%
Self-caught	4%	2%	0%	1%	3%	10%	2%	2%	9%	0%
Gift	3%	2%	2%	3%	3%	2%	6%	1%	2%	2%
Institution	0%	1%	0%	1%	0%	5%	1%	0%	0%	0%
Bar, sports, rec facility	0%	0%	1%	0%	0%	0%	1%	0%	0%	0%
Soup kitchen, com food prog	0%	1%	1%	1%	0%	1%	3%	1%	0%	1%
Other	4%	5%	3%	10%	4%	5%	4%	6%	0%	3%
Don't know	1%	0%	0%	0%	0%	0%	0%	0%	0%	0%
TOTAL	100%	100%	100%	100%	100%	100%	100%	100%	100%	100%

Table S4. Usual fish consumption rates (g/day raw weight, edible portion) of total fish and shellfish. 50th percentile estimates (range: 95% CI). [2].

**Geographic Area**	**Adults 21 yr and older**	**Youth <21 yr**
**Region**		
Northeast	23.9 (20 - 28.7)	5.7 (4.1 - 7.8)
Midwest	12.9 (10.6 - 15.6)	3.3 (2.5 - 4.3)
South	17.6 (15.1 - 20.4)	5.7 (4.2 - 7.7)
West	20 (17.1 - 23.4)	5.9 (4.1 - 8.7)
**Coastal Status**		
Noncoastal	15.9 (13.7 - 18.5)	4.5 (3.5 - 5.7)
Coastal	20.9 (18.4 - 23.7)	5.9 (4.7 - 7.4)
**Coastal/Inland Region**		
Pacific	22.1 (18.2 - 26.7)	5.9 (4.3 - 8.1)
Atlantic	24.5 (20.7 - 28.9)	7.2 (5.4 - 9.6)
Gulf of Mexico	19 (15.2 - 23.8)	7 (4.3 - 11.5)
Great Lakes	14.6 (12.1 - 17.5)	3.9 (2.9 - 5.2)
Inland Northeast	22.1 (17.5 - 28)	5.1 (3.6 - 7.2)
Inland Midwest	12.4 (10.1 - 15.1)	3.1 (2.3 - 4.1)
Inland South	15.6 (13.1 - 18.4)	4.9 (3.7 - 6.4)
Inland West	18.4 (15.1 - 22.5)	6.0 (3.5 - 10.1)

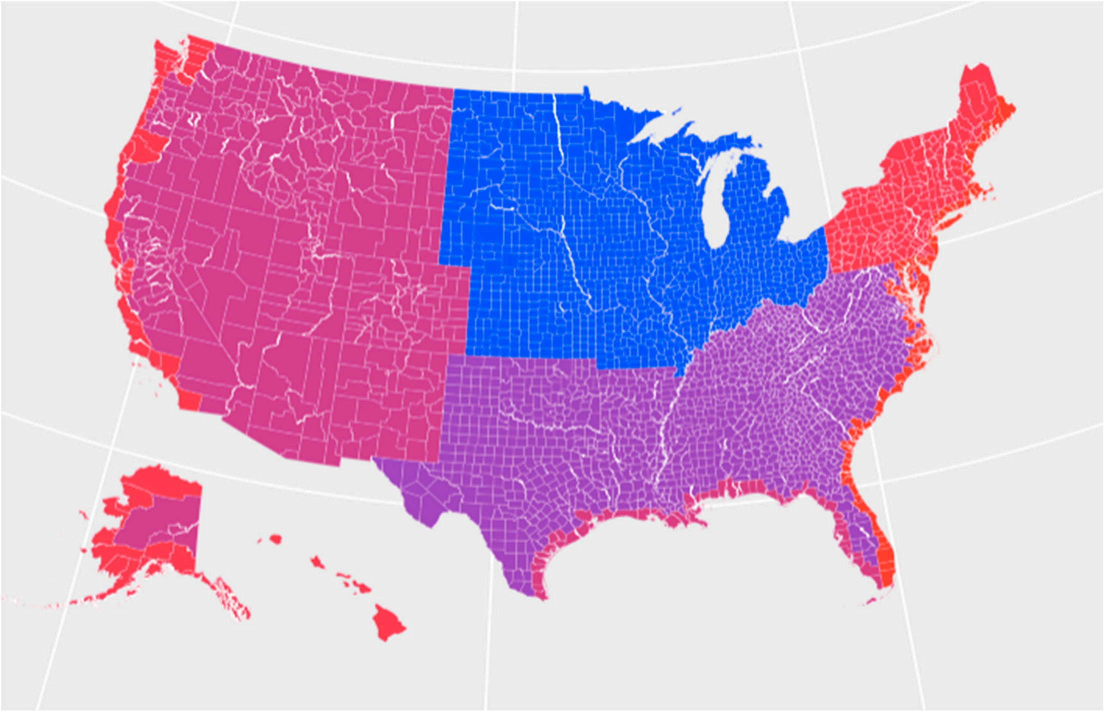

Figure 1. Seafood consumption rates (g/d, raw weight, edible portion) by county for each coastal/inland region described in Table S4.

References

1. Lin, B.-H.; Anekwe, T.D.; Buzby, J.C.; Bentley, J. *US Food Commodity Availability by Food Source, 1994–2008*; US Department of Agriculture, Economic Research Service: Washington, DC, USA, 2016.

2. EPA. *Estimated Fish Consumption Rates for the U.S. Population and Selected Subpopulations (NHANES 2003–2010)*; EPA-820-R-14-002; U.S. Environmental Protection Agency Report an Environmental: Washington, DC, USA, 2014.
